# Supplementary material for: Diabetic Glycation of Human Serum Albumin Affects Its Immunogenicity
Source: Biomolecules. 2024 Nov 23;14(12):1492. doi: 10.3390/biom14121492 (PMC11673269; doi:10.3390/biom14121492)

Raw image Western blots

Disclaimer: For some of the blot multiple samples or conditions were tested which was not part of the main manuscript. However, since we are mandated to show RAW data, here are the blots in full, which includes not-relevant samples/blots as well.

RAGE Western blot (left)

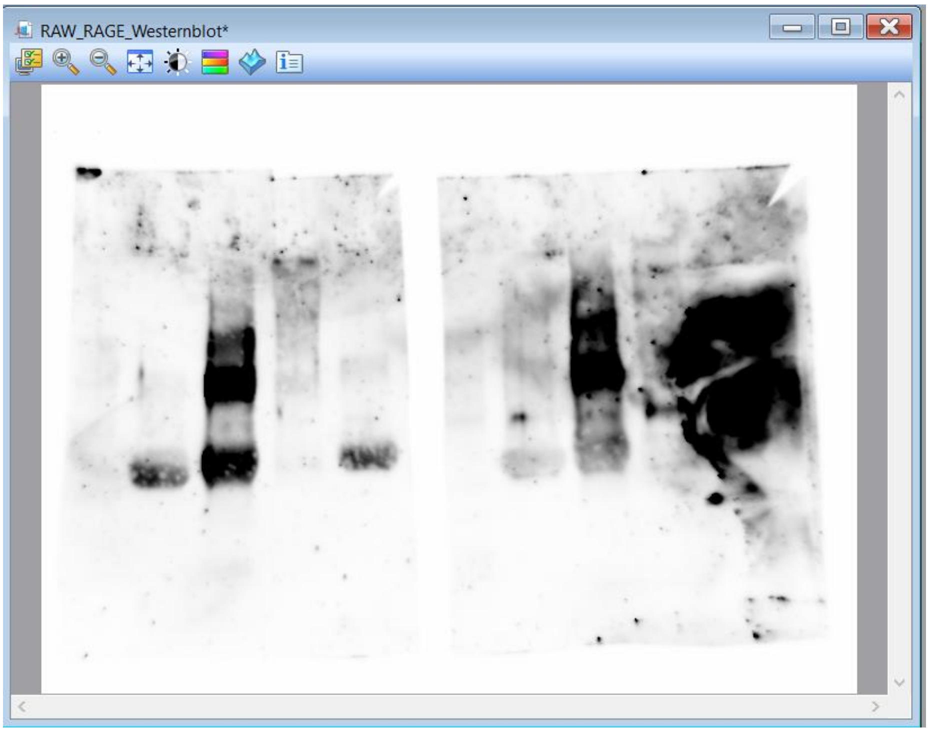

Phospho-NFkB P65 THP-1 sample 1

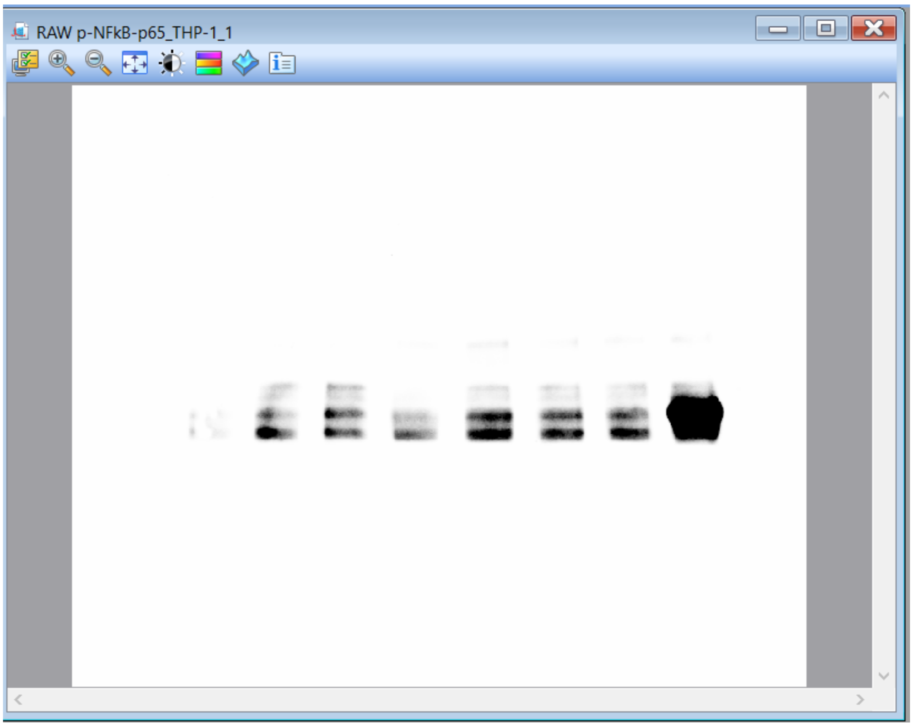

Phospho-NFkB P65 THP-1 sample 2 (lower blot)

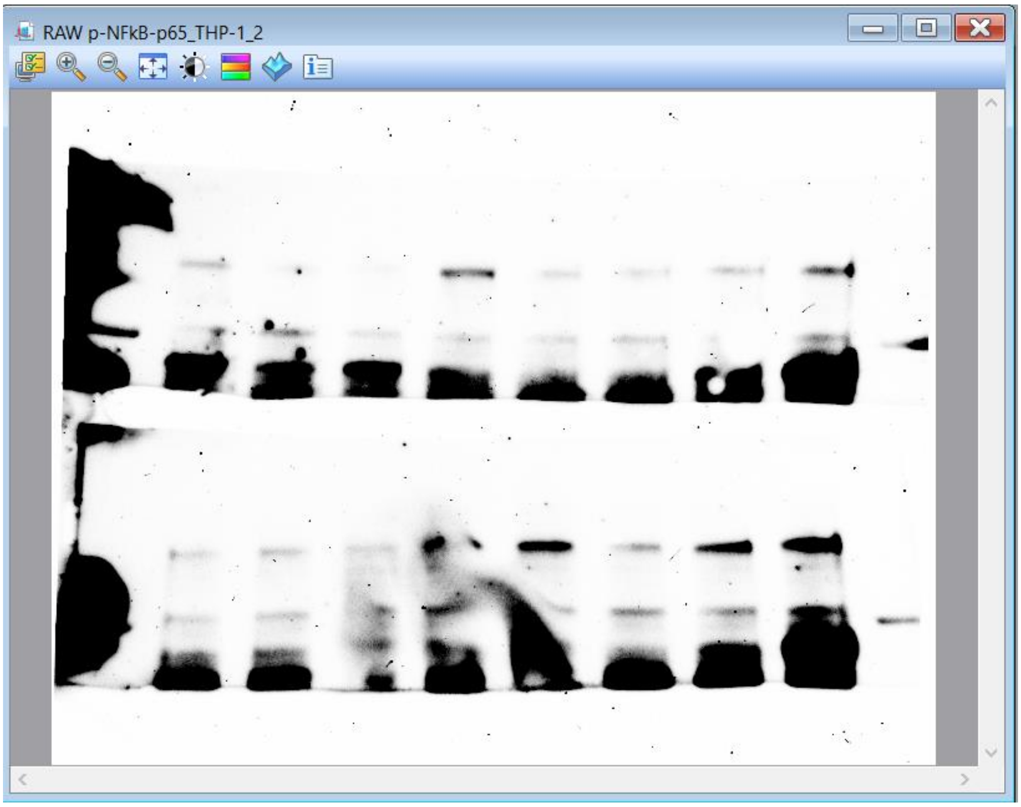

Beta-actin THP-1 sample 1

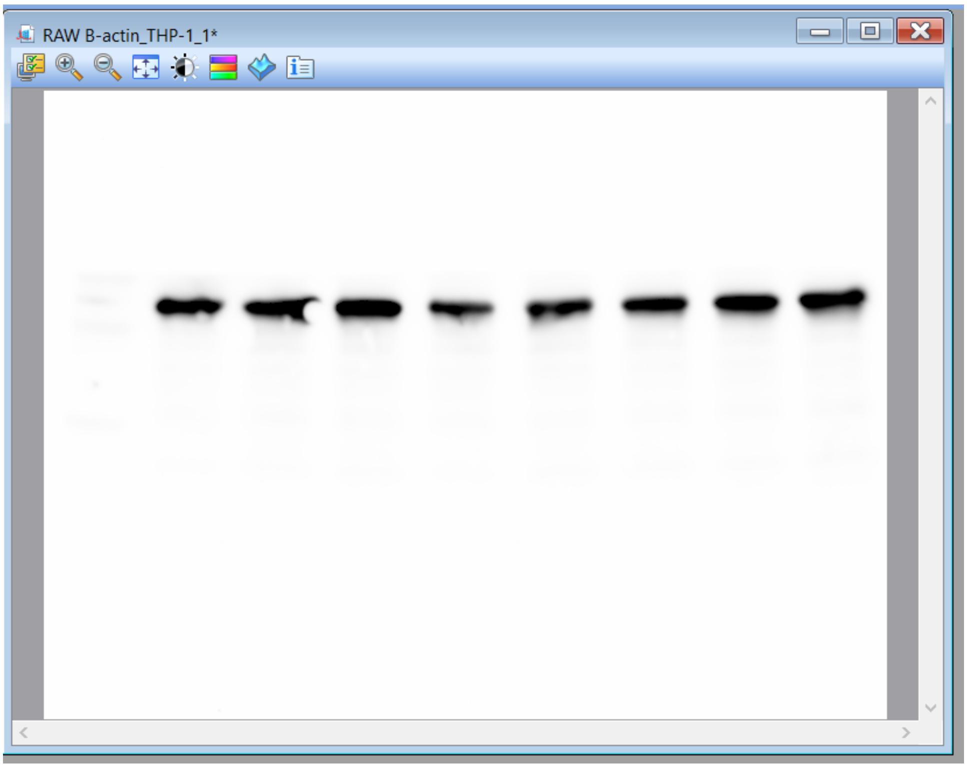

Beta-actin THP-1 sample 2 (lower blot)

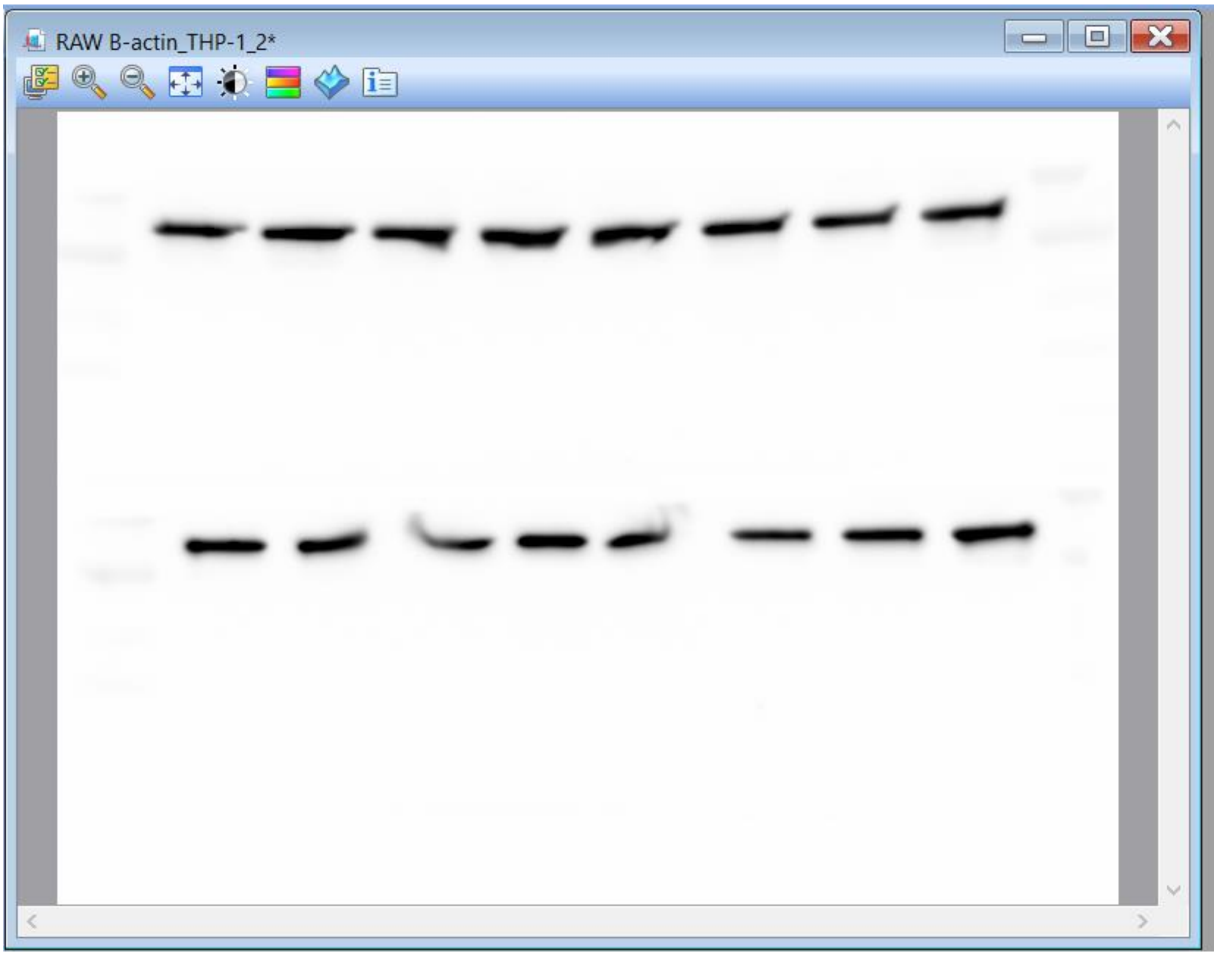

Supplement: Supplementary file 1 [file biomolecules-14-01492-s001.zip › biomolecules-3265008-Supplementary figure s10.pdf]
