# Supplementary material for: Diabetic Glycation of Human Serum Albumin Affects Its Immunogenicity
Source: Biomolecules. 2024 Nov 23;14(12):1492. doi: 10.3390/biom14121492 (PMC11673269; doi:10.3390/biom14121492)
Supplement: Supplementary file 1 [file biomolecules-14-01492-s001.zip › biomolecules-3265008-supplementary-figure s1-s9.pdf]

Supplementary Table 1

Table S1. List of primer sets used in this study.

**Genes**	**Forward**	**Reverse**
PUM1	TGAGGTGTGCACCATGAAC	CAGAATGTGCTTGCCATAGG
IL-1B	TTCGACACATGGGATAACGAGG	TTTTTGCTGTGAGTCCCGGAG
IL-8	CTGATTTCTGCAGCTCTGTG	GGGTGGAAAGGTTTGGAGTATG
TNFa	CTT-CTG-CCT-GCT-GCA-CTT-TG	GTC-ACT-CGG-GGT-TCG-AGA-AG
RAGE	GCT-TGG-AAG-GTC-CTG-TCT-CC	CAC-GGA-CTC-GGT-AGT-TGG-AC
CD86	CTGCTCATCTATACACGGTTACC	GGAAACGTCGTACAGTTCTGTG

Supplementary Figure S1

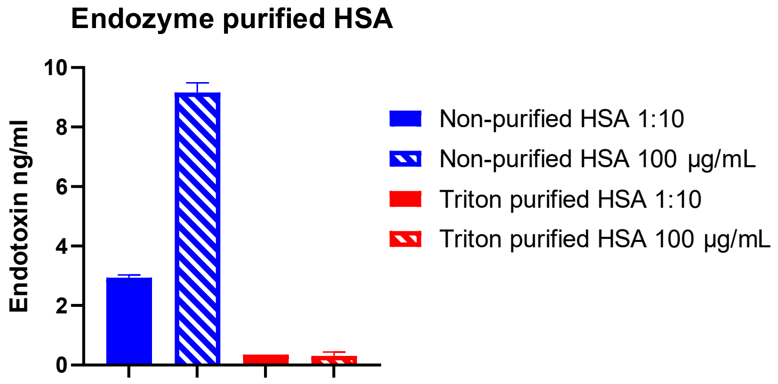

Figure S1. Detection of endotoxin in HSA purchased from Sigma before and after Triton purification.

Supplementary Figure S2

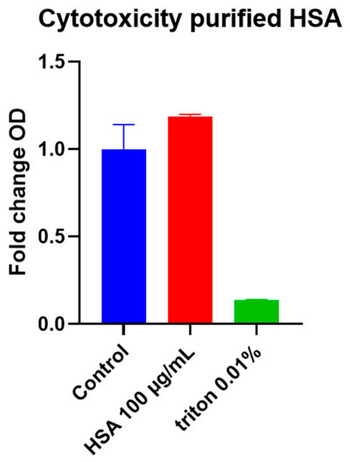

Figure S2. Cytotoxicity assay with purified HSA.

Supplementary Figure S3

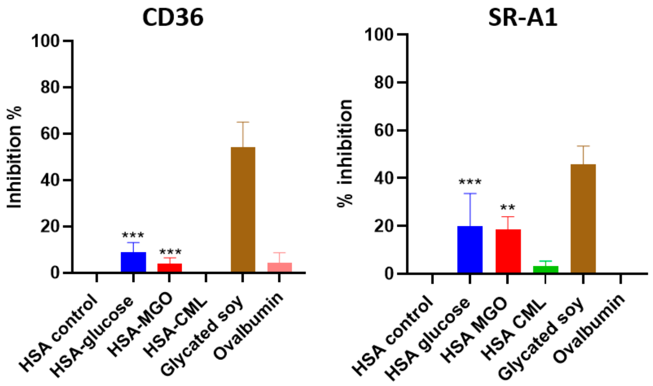

Figure S3. Binding of glycated HSA to SR-A1 and CD36. Significant differences analyzed with One-way ANOVA (GraphPad Prism); ** p < 0.01, *** p < 0.0001.

Supplementary Figure S4

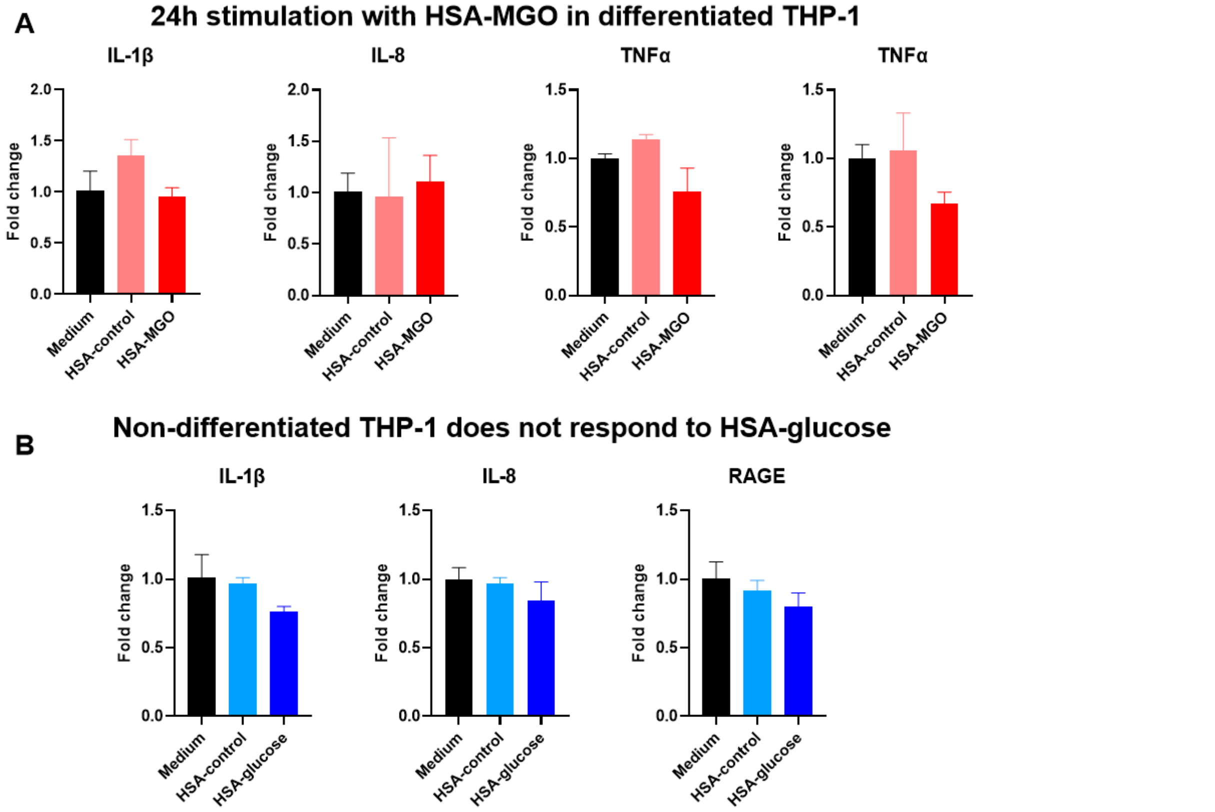

Figure S4. Optimization of THP-1 experiments: Stimulation of THP-1 macrophages with HSA–MGO for 24 h did not result in any effect (**A**), and non-differentiated THP-1 does not respond to 24 h HSA–glucose stimulation (**B**).

Supplementary Figure S5

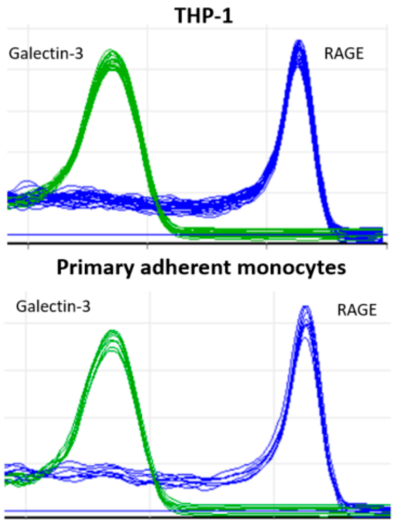

Figure S5. RAGE and Galectin-3 gene expression are expressed on THP-1 and primary monocytes.

Supplementary Figure S6

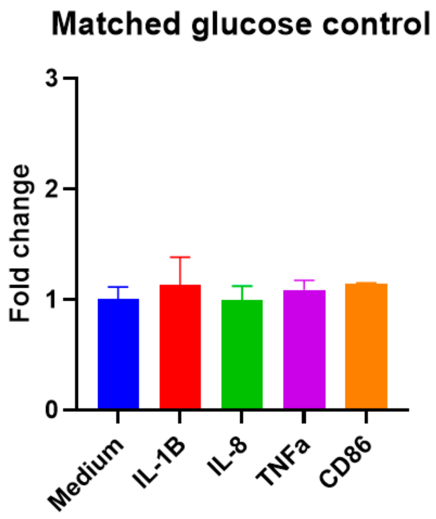

Figure S6. Glucose in buffer control. THP-1 was stimulated with glycated HSA, and inflammatory response was measured on qPCR. Here, we show that glucose-matched control did not impact the cells.

Supplementary Figure S7

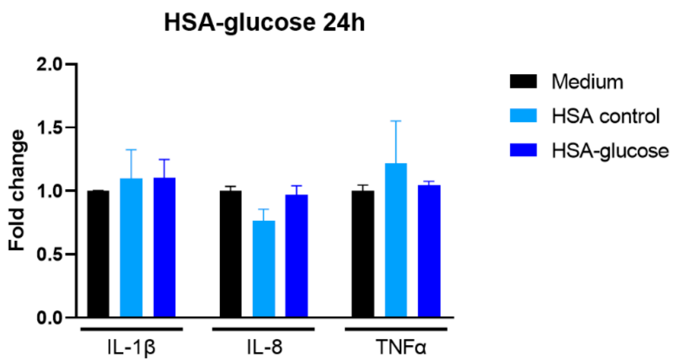

Figure S7. No measurable increase in cytokines was observed after 24 h of stimulation with glycated HSA. For choosing a timepoint we tested both stimulation with 3 h and 24 h, and inflammatory responses were found at 3 h (Figure 6) but not 24 h.

Supplementary Figure S8

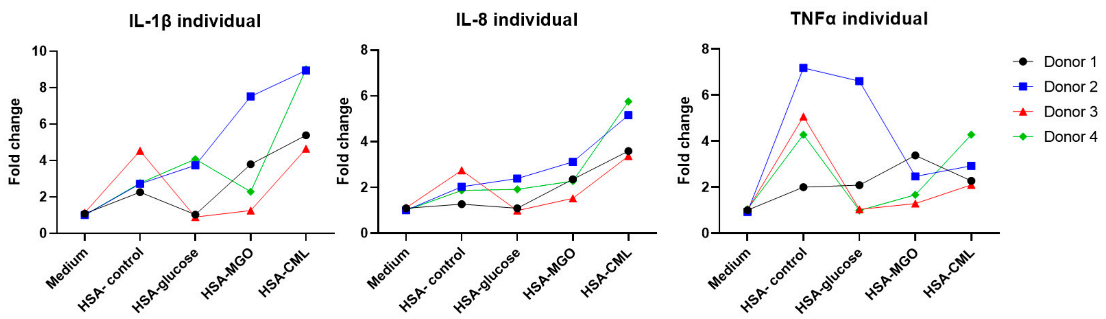

Figure S8. Graph depicting individual donor responses and variation between donors.

Supplementary Figure S9

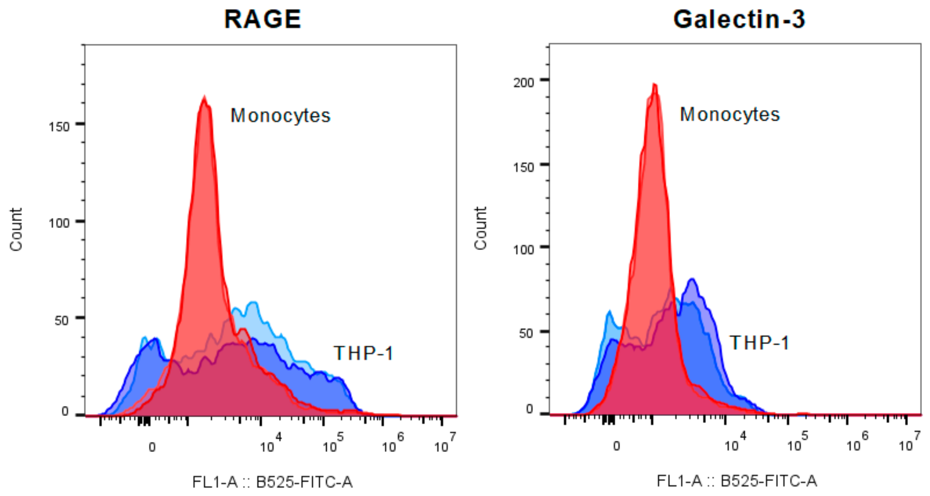

Figure S9. MFI graphs of RAGE and Galectin-3. Red color are primary monocytes, blue color are THP-1 macrophages.
